# PathwayEmbed: a computational tool to quantify intracellular signaling transduction states from transcriptomic data

**DOI:** 10.1093/bioinformatics/btag346

**Published:** 2026-05-28

**Authors:** Yaqing Huang, Sharon Gerecht, Themis Kyriakides, Micha Sam Brickman Raredon

**Affiliations:** Department of Pathology, Yale University, New Haven, CT 06520, United States; Vascular Biology and Therapeutics Program, Yale University, New Haven, CT 06520, United States; Biomedical Engineering, Duke University, Durham, NC 27705, United States; Biomedical Engineering, Duke University, Durham, NC 27705, United States; Department of Pathology, Yale University, New Haven, CT 06520, United States; Vascular Biology and Therapeutics Program, Yale University, New Haven, CT 06520, United States; Vascular Biology and Therapeutics Program, Yale University, New Haven, CT 06520, United States; Department of Anesthesiology, Yale School of Medicine, New Haven, CT 06520, United States; Program in Translational Biomedicine, Biological and Biomedical Sciences, Yale University, New Haven, CT 06520, United States

## Abstract

**Motivation:**

Intracellular signaling pathways regulate essential cellular functions and orchestrate complex biological processes, yet their dynamic activity remains challenging to quantify with precision. Advances in single-cell omics enable pathway activity inference at the transcriptional level; however, existing computational tools often overlook mechanistic features of signaling networks, failing to formally treat the expected directionality of transcriptional change due to signal transduction. To address this technological gap, we have engineered PathwayEmbed, an R-based computational framework for estimating intracellular signal transduction states from single-cell transcriptomic datasets.

**Results:**

PathwayEmbed integrates KEGG pathway information with perturbation-derived RNA sequencing data to assign directional coefficients that capture gene-specific transcriptional responses to pathway activation, repression, and/or signal transduction. These coefficients, in combination with the input data, are used to compute hypothetic ON/OFF range for each pathway. Each cell is then mapped to a specific location between these ON/OFF states, and activity scores are then computed based on the distances to these reference states, providing a continuous and interpretable measure of signaling activity at single-cell resolution. This framework enables robust visualization and quantitative comparison of pathway activity across cell populations. Applied to spatial transcriptomic data, PathwayEmbed captures spatial variation in signaling transduction states and allows comparisons at both temporal and spatial scale. The framework takes tabular data as input and is broadly compatible with established single-cell analysis workflows, supports user-defined pathway ground-truths, and offers a flexible, mechanistically informed approach for quantifying and comparing intracellular signaling activity in a wide variety of contexts.

**Availability:**

PathwayEmbed is an open-source R software under academic free license, and it is available at https://github.com/raredonlab/PathwayEmbed. Use-case vignettes are available at https://raredonlab.github.io/PathwayEmbed/.

## 1 Introduction

Intracellular signaling pathways are fundamental to cellular decision-making, coordinating responses to environmental cues and maintaining homeostasis across diverse biological contexts. Dysregulation of these pathways contributes to the pathogenesis of numerous diseases, including cancer, fibrosis, and autoimmune disorders. Accurate quantification of dynamic signaling states is therefore essential for elucidating disease mechanisms, improving diagnosis, and guiding the development of targeted therapies.

Advances in high-throughput sequencing technologies have enabled transcriptional-level inference of intracellular pathway activity (Zhang *et al.* 2020). While gene expression profiles offer a rich resource for this purpose, interpreting accurate pathway activities from such large-scale data remains challenging. Commonly used approaches, for example, Seurat’s AddModuleScore (Tirosh *et al.* 2016), calculate a module score using the mean expression of predefined gene sets subtracted by control gene sets. AUCell (Aibar *et al.* 2017) and GSVA (Hanzelmann *et al.* 2013) use a gene-ranking approach to determine the correlation between pathways and cells based on the area under ranked genes. Each of the above methods are straightforward to implement but treat pathways as undirected collections of genes. In other words, genes in given pathways are regarded as upregulated in all conditions. This overlooks critical biological features, including the activating or inhibitory roles of individual molecules, feedback regulation, and differential weighting based on mechanistic relevance. To be specific, transcriptional changes in response to external stimuli might not always be the same at the feature level, depending on the role of corresponding proteins involved. Simple aggregation of gene expression without encoding this directionality may therefore fail to reflect the true net transduction state of signaling pathways, particularly in heterogeneous or perturbed systems. Several existing tools incorporate aspects of regulatory logic or directionality and demonstrate improvement in interpretation of multi-omic data. For example, Signaling Pathway Impact Analysis (Tarca *et al.* 2009) incorporates KEGG interaction topology to identify perturbed pathways but is restricted to population-level analysis and does not produce per-cell activity scores. PROGENy (Schubert *et al.* 2018) and VISION (DeTomaso *et al.* 2019) are applicable to single-cell data and both support signed gene signatures. PROGENy (Schubert *et al.* 2018) derives continuous, signed gene weights by fitting a linear model across a large compendium of bulk RNA-seq perturbation experiments, then applies these weights as a fixed matrix multiplication to score each cell. VISION (DeTomaso *et al.* 2019) computes per-cell weighted expression sums from signed signatures and uses local autocorrelation statistics on the single-cell similarity manifold to identify biologically coherent patterns of variation. However, both approaches generate summary scores with no inherent bounds that based on a predetermined external reference rather than the signaling landscape of the dataset being analyzed.

Inspired by these advances, we developed PathwayEmbed, an R-based computational framework to quantify intracellular signaling transduction activity from transcriptomic data. The key distinction from existing tools is our use of a geometric, reference-state embedding approach: instead of aggregating expression values or computing scores relative to a fixed external population, PathwayEmbed derives its reference anchors from the observed expression extremes of pathway genes within the input data itself, adapted to the biological context at hand. PathwayEmbed accepts raw, normalized, or scaled expression data, offering flexibility for different users and processing workflows. Benchmarking analyses demonstrate both reliability and scalability with increasing dataset size. The resulting pathway scores can be readily visualized with ggplot2 (Wickham 2016) or integrated into established single-cell analysis platforms, including Seurat (Hao *et al.* 2024), to facilitate customized downstream analyses.

## 2 Approach

**Figure 1:**
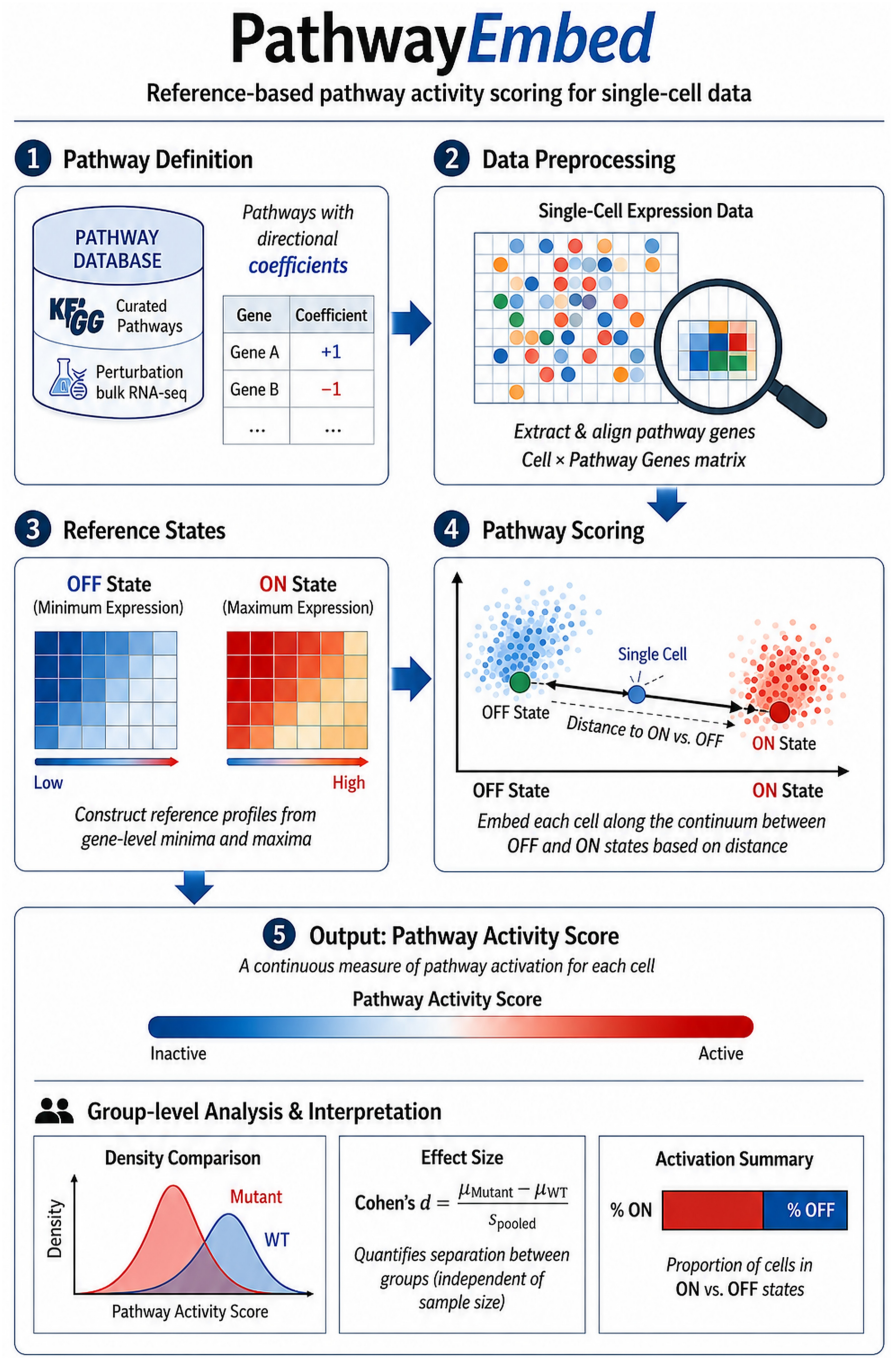
Overview of PathwayEmbed.

PathwayEmbed is an R package for inferring intracellular signaling transduction states from transcriptomic data using a reference-based embedding framework. The framework operates on one pathway at a time, using as input a single-cell expression matrix (cells × genes) and a focused pathway model (pathway genes × coefficients), to generate pathway activation scores for each cell. Each gene is assigned a directional coefficient representing its expected transcriptional response during pathway activation. Directionality is informed by a data-driven perturbation-derived transcriptional data, and tools are provided to allow users to leverage priors in their determination of expected coefficients. In the current implementation, coefficients are discretized to +1 or −1 to encode upregulation or downregulation, respectively; however, the framework is flexible and allows user-defined coefficients to reflect alternative pathway models or context-specific regulatory weights if this is desired. The resulting pathway coefficient table can be viewed using ListPathway() and directly loaded via the LoadPathway() functions. Given an input expression matrix, PathwayEmbed first extracts pathway-associated genes and optionally performs row-wise scaling using DataPreProcess(). Rather than applying weighted aggregation, the method constructs pathway-specific reference states using PathwayMaxMin(). Two high-dimensional reference points, pathway.on and pathway.off, are generated to define the full extent of pathway-transduction space. Briefly, the ON reference takes the expression value that maximizes the signal in the direction indicated by its coefficient, that is, the maximum observed expression for positively signed genes (+1) and the minimum observed expression for negatively signed genes (−1). The OFF state is constructed with the opposite logic. These ON/OFF values define the observed possible expression range for pathway-relevant genes within the input dataset and can be subsequently used for normalization. Each cell is then positioning within this pathway-defined space by computing its distance to the ON and OFF reference states using a user-defined metric (Manhattan distance by default, or alternatively Euclidean distance). Pathway activity is quantified as the normalized relative distance between these two reference states [score = distance_to_OFF/(distance_to_ON + distance_to_OFF)], yielding a continuous score bounded between inactive (OFF-like) and active (ON-like) states. Importantly, this formulation defines pathway activity as a relative position within a data-adaptive transduction space, rather than an absolute value derived from predefined signatures. Finally, resulting scores can be merged with cell metadata to enable comparisons for various scientific purposes (Fig. 1). PathwayEmbed is implemented in R (≥ 3.5) and depends on dplyr (Wickham *et al.* 2025), tidyverse (Wickham *et al.* 2019), matrixStats (Bengtsson 2017), ggplot2 (Wickham 2016) and effsize (Torchiano 2020). Detailed function descriptions, biological assumptions, and recommended best practices are provided in the Supplementary Material. The package, along with example data and usage documentation, is available at https://raredonlab.github.io/PathwayEmbed/.

## 3 Application

### 3.1 Advantages of PathwayEmbed

PathwayEmbed provides a novel framework for quantifying and visualizing pathway activation states at single-cell resolution by integrating curated pathway molecule coefficients with gene expression data. Unlike many existing pathway analysis tools that focus solely on gene set enrichment or overall gene relevance, PathwayEmbed incorporates the directional influence (activating/inhibitory, produced/degraded) of each pathway component according to information from prior perturbation data. This enables a more mechanistic and accurate interpretation of pathway dynamics, which we have validated using known perturbation datasets, such as Ctnnb1 enhancer and Notch knock out single-cell RNA-seq experiments (Hua *et al.* 2024) (Supplementary Figs. 5 and 6). Pathway coefficients are fully customizable (an example is provided in the vignettes detailing appropriate methods), allowing researchers to adapt pathway definitions to specific biological contexts or experimental conditions, which is frequently necessary during applied biological analysis. Moreover, PathwayEmbed supports input of either raw or normalized counts or Seurat object, providing flexibility for diverse preprocessing workflows (vignette demonstration in Toy Set: https://raredonlab.github.io/PathwayEmbed/articles/examples_updated.html).

By normalizing molecular distances relative to hypothetical “On” and “Off” states, the resulting scores are easily interpretable compared to other decomposition approaches (Fan *et al.* 2016, Tomfohr *et al.* 2005). This design provides several advantages. By encoding directionality through reference-state construction rather than direct weighting, PathwayEmbed preserves interpretability while capturing gene-specific regulatory behavior. The use of dataset-derived extrema enables adaptation to diverse biological contexts, including temporal and spatial transcriptomic data. However, as pathway activity is defined relative to dataset-specific reference states, it is important to note that scores are inherently context-dependent. As such, comparisons across independent datasets may require additional normalization or calibration. Overall, the pairwise distance method approach simplifies interpretation, producing continuous activation scores that can be visualized using density plots or integrated with cell metadata for downstream comparative analyses.

### 3.2 Compute net transduction states of individual cells

To illustrate its utility, we first generated a synthetic toy dataset containing two populations of cells expressing Wnt pathway molecules and other random genes (Supplementary Figs. 2–4). Using normalized values, we calculated the net Wnt transduction score for individual cells and visualized the density distribution with the embedded plotting function. The shift in Wnt transduction states between predefined wild-type (WT) and mutant groups remained highly consistent across different computational batch sizes. Effect size analysis using Cohen’s *d* revealed a large separation between the two populations distributions, with minimal correlation between Cohen’s *d* values and the number of run iterations, indicating the robustness of the approach for detecting differences in signaling transduction states. We further applied PathwayEmbed to public scRNA-seq datasets from perturbation experiments (Remark *et al.* 2023, Hua *et al.* 2024), and the results aligned with the expected biological interventions, including gene knockout and aging (Supplementary Figs. 5–7). Detailed descriptions of each experiment are provided in the Supplementary Data.

### 3.3 Map spatial distribution of signaling activation

We further applied PathwayEmbed to mouse embryonic spatial transcriptomics data (Chen *et al.* 2022) to examine Wnt, Notch, TGF-β, Hippo, and HIF-1α signaling transduction states. By integrating pathway activation scores with spatial coordinates and evaluating the resulting patterns with Moran’s *I*, we observed clear spatial segmentation of signaling states across embryonic regions during development (Supplementary Figs. 8 and 9). Density plots and spatial feature plots enabled direct comparison of relative transduction states across developmental time points (Supplementary Figs. 8 and 9). A detailed vignette demonstrating this workflow is available online at: https://raredonlab.github.io/PathwayEmbed/articles/spatial_pathway.html.

## 4 Conclusion

PathwayEmbed provides a flexible and mechanistically informed framework for quantifying pathway activity at single-cell resolution, offering insights that complement and extend beyond conventional gene set enrichment methods. Its core innovation is the geometric positioning of each cell between data-derived ON and OFF reference states constructed from a perturbation-informed pathway gene set, producing a continuous and interpretable transduction score without reliance on fixed external reference panels. This design captures pathway-specific directional structure that adapts to the biological context of each dataset. Continued refinement of molecular coefficients, coupled with integration of experimental validation, will further improve accuracy and broaden applicability, enabling more precise and biologically grounded analyses of intracellular signaling in single-cell omics.

## Acknowledgement

The authors used artificial intelligence (AI) tools, including ChatGPT (OpenAI), for grammar and language editing and for assistance with figure revision.

## Supplementary Material

btag346_Supplementary_Data

## Data Availability

All data needed to evaluate the conclusions in the paper are included in the manuscript and package and can be accessed publicly.
