# Supplementary material for: PathwayEmbed: a computational tool to quantify intracellular signaling transduction states from transcriptomic data
Source: Bioinformatics. 2026 May 28;42(6):btag346. doi: 10.1093/bioinformatics/btag346 (PMC13292150; doi:10.1093/bioinformatics/btag346)
Supplement: btag346_Supplementary_Data [file btag346_supplementary_data.docx]

# **PathwayEmbed: A Computational Tool to Quantify Intracellular Signaling Transduction States from Transcriptomic Data.**

Yaqing Huang ^1,2,3^, Sharon Gerecht^3^, Themis Kyriakides^1,2^, and Micha Sam Brickman Raredon* ^2,4,5^

Department of Pathology, Yale University, New Haven, CT 06520, USA [1]

Vascular Biology and Therapeutics Program, Yale University, New Haven, CT 06520, USA [2]

Biomedical Engineering, Duke University, Durham, NC 27705, USA [3]

Department of Anesthesiology, Yale School of Medicine, New Haven, CT 06520, USA [4]

Program in Translational Biomedicine, Biological and Biomedical Sciences, Yale University [5]

*Corresponding author:

Prof. Micha Sam Brickman Raredon, [michasam.raredon@yale.edu](mailto:michasam.raredon@yale.edu)

## **Software Description**

**Overview of PathwayEmbed**

The tool leverages a custom-curated pathway coefficient table of signaling molecules and mechanistic coefficients, integrated with distance-based embedding strategy to produce biologically meaningful pathway activity representations at the sample or single-cell level (**Supplementary Fig.1**).

**Supplementary Figure 1. Overview of PathwayEmbed** (A) Schematic representation of the PathwayEmbed mechanism. (B) Workflow of the PathwayEmbed package, outlining key functions included in the package.

**Mechanistic Pathway Database Curation**

Central to PathwayEmbed theory is that the changes of individual transcript species should not be treated equally during pathway activation. Instead, pathway coefficients should reflect both the molecular components and regulatory architecture of intracellular signaling pathways. To achieve this, each gene or molecule in the pathway coefficient table is assigned a directional coefficient that encodes its expected transcriptional response (e.g., upregulation or downregulation) during pathway activation, thereby capturing gene-specific contributions to signal transduction.

Pathway coefficients are constructed by integrating established biological databases with perturbation-derived transcriptional data. Core pathway gene sets are obtained from curated resources such as KEGG, which define the canonical components of signaling pathways (e.g., WNT, TGFB, NOTCH, HIF1A, HIPPO). Within each pathway, genes are further organized into functional categories reflecting their roles in the signaling cascade (e.g., Ligands, Receptors, Transcriptional Targets). Each gene entry is annotated with both human (HGNC) and mouse (MGI) gene symbols, enabling direct application to datasets from either species without additional ortholog translation steps. These curated gene sets and coefficients provide the structural backbone of each pathway model.

Perturbation datasets have been selected with the goal of approximating physiological signaling states. Specifically:

- Experiments involving ligand stimulation are prioritized
- Human and mouse datasets are included to ensure broad applicability
- Experiments involving genetic manipulation (e.g., knockouts or overexpression systems) were excluded, as these may alter baseline regulatory architecture and confound interpretation of pathway dynamics

Detailed metadata describing each dataset incorporated thus far (including GEO accession, experimental condition, cell type, species, and number of genes) have been stored in a summary table accessible via the ListPathway() function, allowing users to select pathway coefficient table most appropriate for their biological context. For each pathway, differential expression analysis was performed to quantify gene-level responses to pathway activation. Genes were assigned directional coefficients reflecting their transcriptional behavior, with +1 denoting upregulation and −1 denoting downregulation upon pathway activation. This approach enables encoding of both directionality and gene-specific contributions to signaling dynamics. When multiple conditions or timepoints are available, pathway coefficient tables can be constructed in a condition-specific manner (e.g., early vs. late activation), enabling representation of dynamic signaling behavior. The resulting pathway database is organized as a collection of gene–coefficient tables, where each pathway-condition pair is represented by Molecules and Coefficients. Additional annotations (e.g., species mapping, differential expression metrics, and temporal features such as time-regression slopes) are retained to support downstream analyses and cross-species compatibility. An example of this construction process is provided in the vignette using the TGFβ pathway.

A preconstructed pathway coefficient excel is provided within the package and can be modified or extended by users to reflect alternative biological models, experimental systems, or cell-type specific contexts. The pathway information is loaded into the R environment via the LoadPathway() function, which parses the spreadsheet into structured pathway objects. These objects retain molecule-relationship mappings and coefficient annotations, which are subsequently used to guide the embedding and downstream analyses.

**Pathway Score Calculation**

Following pathway coefficient construction, PathwayEmbed quantifies pathway activity at the single-cell level by embedding each cell relative to pathway-specific reference states derived from the input data via the following three steps:

**Data preprocessing.** The first step prepares the expression data for pathway-specific scoring. Expression data can be provided either as a gene-by-cell matrix or as a processed Seurat object. The DataPreProcess() function filters the expression matrix to pathway genes and optionally performs row-wise scaling (z-score normalization), allowing users to control whether relative or absolute expression values are used in downstream analysis. The output is a pathway-gene-by-cell matrix of filtered and optionally scaled expression values.

**Reference state construction.** The central innovation of PathwayEmbed is the construction of two high-dimensional reference profiles derived directly from the input dataset. PathwayMaxMin() generates a hypothetical ON and OFF reference state, representing maximal and minimal expected pathway activation, respectively, by combining the extremal expression values of each molecule in the global dataset. Specifically, for genes with a coefficient of +1 (expected to be upregulated during pathway activation), the ON state takes the maximum observed expression value for that gene and the OFF state takes the minimum. For genes with a coefficient of −1 (expected to be downregulated during activation), this logic is inverted: the ON state takes the minimum observed value and the OFF state takes the maximum. The resulting theoretical activation range of the pathway allows an initial assessment of the dataset and serves as a critical scale for subsequent embedding.

**Per-cell distance-based scoring.** After determining the scope within which individual cells are compared, ComputeCellData() will then compute distance of an individual cell to the Pathway ON and OFF states using a user-specific metric. The per-cell pathway activity score is then reported as score = d_OFF / (d_ON + d_OFF), yielding a value bounded between 0 and 1. The default distance methods chosen is “Manhattan”, which sums absolute difference, for its straightforward use in high-dimensional data and less sensitivity to outliers. Alternatively, Euclidean distance can be applied to capture straight-line relationships in expression space. These distances quantify the relative similarity of each cell to the ON and OFF reference states.

To visualize the dissimilarities, normalization within the global range is applied and returns values representing the position of each cell in the transduction space, bounded by the hypothetical ON (active) and OFF (inactive) states. Consequently, pathway scores reflect relative signaling activity within the context of the input dataset, rather than absolute pathway activation levels.

**Comparisons and Visualization**

Pathway transduction state scores for each cell obtained from ComputeCellData() can be treated as cell metadata and added to the original single cell object for statistical analysis and visualizations. The function PreparePlotData() in the package also offers to integrate metadata extracted from the original (Seurat) scRNA seq object to the score lists. By choosing the “group” variables, it allows easier comparisons between different identities. In addition to the normalized pathway score (bounded between 0 and 1), PreparePlotData() generates a z-scored version of the pathway activity (scale), which is centered across all cells. By doing this, a dashed line with x = 0 represents the mean pathway transduction status in the follow-up density plot generation using “PlotPathway()” function. This plotting function is built based on ggpolot2 and cowplot, which allows customized color palettes, titles, and annotations, enabling flexible visualizations of pathway activity differences. In addition, users may directly use ggplot2 to generate custom visualizations from the output of PreparePlotData(). Lastly, a CalculatePercentage() function is provided in the package to quantify the proportion of cells in active versus inactive states to provide group-level comparison. For two-group comparison, a Cohen’s d and a Wilcoxon rank-sum test p-value will be computed to report both the magnitude and statistical significance of differences between distributions. For multi-group comparisons, a Kruskal–Wallis test is performed, and pairwise Wilcoxon tests with multiple-testing correction are provided. Together, these functions enable comprehensive comparison of pathway activity distributions across cell populations, combining visualization, effect size estimation, and statistical inference.

## **Software Validation**

PathwayEmbed captures pathway activation differences in a controlled dataset

### To evaluate PathwayEmbed’s performance, we generated a synthetic single-cell RNA-seq dataset simulating Wnt signaling activation. The dataset consisted of two groups, wild-type (“WT”) and mutant (“Mutant”), with 1,000 cells per group. Simulated gene expression counts were generated from a Poisson distribution. Genes of molecules involved in activated Wnt Pathway transduction, including receptor Lgr5, transcriptional Tcf/Lef complex, downstream transcription of Myc, and Axin2 were synthetically ‘upregulated’ in the Mutant group by adding small random integer offsets. Together with additional genes from unrelated pathways, the dataset comprised 100 genes in total. The resulting count matrix (synthetic_test_matrix_100) was converted to a Seurat object and stored in the package (synthetic_test_object_100) with genotype metadata, providing a controlled framework for systematic evaluation of pathway activity inference and benchmarking against existing methods.

### To establish WNT pathway coefficients aligning with ligand-based signal transduction, we queried GEO for bulk RNA-seq datasets capturing canonical WNT pathway activation via ligand stimulation in human cell systems, excluding experiments involving genetic manipulation. A time-course dataset (GSE103175) in which human embryonic stem cells (H1 hESCs) were treated with recombinant WNT3A protein and profiled at 12, 24, and 48 hours relative to an untreated baseline was selected (Huggins et al. 2017), as recombinant ligand treatment closely approximates physiological pathway activation. Given the single-replicate design of this dataset, differential expression was assessed using a limma contrast framework for each timepoint versus baseline, and a gene-wise linear regression slope across all four timepoints was additionally computed to capture temporal trends. Genes upregulated relative to baseline received a coefficient of +1 and downregulated genes received −1, yielding four condition-specific pathway coefficient tables including WNT3A_12H, WNT3A_24H, WNT3A_48H, and WNT3A_SLOPE, each covering approximately 85–90 genes from the KEGG WNT signaling pathway (hsa04310).

### Using condition-specific WNT pathway coefficient tables (12 h, 24 h, 48 h, and slope), PathwayEmbed identified clear differences in pathway activity between the synthetic WT and mutant cells across all conditions, with more pronounced separation observed at early timepoints (12 h and 24 h). For example, at 12 h, approximately 61.7% of mutant cells were classified as ON compared to 41.5% of WT cells, corresponding to a moderate effect size (Cohen’s d ≈ −0.45). These differences were visualized using density plots, violin plots, and waterfall plots, all of which consistently showed a rightward shift in the pathway activity score distribution in mutant cells relative to WT (Supplementary Fig. 2).These results demonstrate that PathwayEmbed captures biologically meaningful differences in ground-truth pathway transduction states and that condition-specific pathway coefficient tables enable more refined evaluation of signaling dynamics.

### PathwayEmbed also supports user-defined pathway coefficient tables for cases where the built-in pathway coefficient table does not cover a pathway of interest or where the user wishes to impose prior biological knowledge directly. To demonstrate this, we constructed a minimal WNT pathway coefficient table comprising 18 genes with manually assigned directional coefficients, including activators (e.g., Lgr5, Ctnnb1, Myc, Axin2, Tcf7) assigned +1 and negative regulators (e.g., Lrp5, Lrp6, Apc, Dvl1) assigned −1, reflecting canonical WNT signaling logic. Applying this customized set of coefficients to the synthetic dataset recovered the expected difference between mutant and WT cells, confirming that PathwayEmbed is flexible and extensible beyond its preconstructed pathway coefficient table (illustrated in the vignette).

###

**Supplementary Figure 2. Demonstration of PathwayEmbed using Synthetic Wnt Mutant Dataset** (A) Output of PathwayEmbed in evaluating the Wnt Mutant Synthetic Dataset based on different pathway coefficient tables and example of visualization using (B) Density Plot, (C) Violin Plot, and (D) waterfall plot.

### ****Null distribution analyses confirm that biological gene selection drives pathway activity signals****

### To assess whether pathway activity scores could arise from gene set size or random variation alone, we constructed a null distribution by sampling random gene sets across a range of sizes and computing their corresponding Cohen’s d values. Specifically, for each gene set size n, random gene sets were sampled from the pool of genes present in the input dataset. A sparse grid of gene set sizes was used, with finer resolution at small sizes (5–50 genes, step = 5) and coarser resolution at larger sizes (60–200 genes, step = 20), while always including the exact size of the real pathway to enable direct comparison.

### For each size, 20 random gene sets were generated. Each gene in the sampled set was assigned a random directional coefficient with equal probability, mimicking the structure of real pathway coefficient tables while removing biological signal. These synthetic pathways were then processed using the full PathwayEmbed pipeline, including preprocessing, reference state construction, distance-based scoring, and group-level comparison. For each random pathway, Cohen’s d was computed between WT and mutant cells.

### The resulting null distribution showed that mean effect sizes were centered around zero across gene set sizes, with increased variability observed for smaller gene sets. At the matched pathway size, the observed effect was not significantly different from the null expectation, indicating that similar effect sizes can arise from random gene sets (Supplementary Fig. 3A).

### In the second analysis, random subsets of genes were drawn exclusively from the genes already present in the 12 h WNT pathway coefficient table, preserving the original coefficient assignments. This tested whether subsets of pathway-annotated genes were sufficient to recover the signal. The within-pathway null analysis further showed that Cohen's d became more stable as more pathway genes were included, indicating that each gene contributes incrementally to the overall signal (Supplementary Fig. 3B).

### Importantly, these findings demonstrate that gene set size alone does not systematically drive pathway activity signals. Instead, biologically meaningful results depend on the selection of functionally relevant genes, emphasizing the importance of pathway curation and coefficient assignment.

###

**Supplementary Figure 3.** Null distribution analysis using (A) random genes and (B) pathway genes. Comparisons with existing methods using (C) AUROC and Cohens’d and (D) Spearman correlation.

### Comparison with existing methods and signal characterization

### We next compared PathwayEmbed with commonly used pathway and expression-based scoring approaches, including PROGENy, AddModuleScore, and simple expression-derived metrics (mean expression and z-scored expression).

### Across methods, expression-based metrics achieved the highest classification performance (AUROC ≈ 0.75) and largest effect sizes (Cohen’s d ≈ 0.95), indicating that the dominant signal in this synthetic dataset is driven by global transcriptional shifts. PathwayEmbed demonstrated moderate performance (AUROC ≈ 0.62; Cohen’s d ≈ 0.38), outperforming AddModuleScore and PROGENy in terms of effect size, but remaining below expression-based baselines (Supplementary Fig. 3C).

### Pairwise correlation analysis further revealed that expression-based metrics were highly correlated with each other, while AddModuleScore also showed strong correlation with mean expression, reflecting its dependence on aggregate expression levels. In contrast, both PathwayEmbed and Progeny exhibited weak correlation with expression-based metrics, indicating that it captures a signal that is partially independent of global transcriptional variation (Supplementary Fig. 3D).

### Together, these results suggest that while global expression dominates signal detection in this dataset, PathwayEmbed captures complementary, pathway-specific structure. This behavior is consistent with its design as a reference-based embedding framework, which models pathway activity relative to mechanistically defined ON and OFF states rather than relying on direct aggregation of gene expression.

### Sensitivity to preprocessing and distance metrics

### We next evaluated the robustness of PathwayEmbed to variations in input data type and distance metrics. Across all tested input including raw counts, CPM-normalized, log-normalized, and restriction to highly variable genes (HVGs) with and without row-wise scaling, PathwayEmbed consistently detected pathway activity differences between mutant and WT cells (Supplementary Fig. 4A). Raw counts yielded the most pronounced separation, producing the largest Cohen's d among all input types (Supplementary Fig. 4B). However, scores derived from raw counts also showed higher correlations with technical covariates (nCount, nFeature, and mean expression) compared to those from normalized inputs, indicating that raw count scores are more susceptible to confounding from differences in library size and sequencing depth (Supplementary Fig. 4C). In contrast, log-normalized and CPM-normalized inputs produced more conservative but technically cleaner scores. Restricting analysis to highly variable genes substantially reduced the observed signal, suggesting that WNT pathway activity in this dataset is distributed broadly across genes rather than concentrated within the variable gene pool. Within each preprocessing type, comparison of scaled and unscaled inputs showed strong agreement in pathway activity scores, indicating that row-wise scaling does not substantially alter relative pathway ranking in this controlled setting, though its effect may be larger in real-world datasets with greater expression heterogeneity. Based on these findings, we recommend log-normalized expression data with row-wise scaling as the default input for PathwayEmbed, balancing detection sensitivity with robustness to technical variation.

### In terms of distance metric, comparison of Manhattan and Euclidean distances revealed highly consistent results across conditions (Supplementary Fig. 4D), indicating that PathwayEmbed is generally robust to the choice of distance metric. Minor differences in score distributions were observed, reflecting expected sensitivity of distance-based methods in high-dimensional spaces.

### In summary, analyses on the synthetic WNT dataset collectively demonstrate that PathwayEmbed: (1) captures biologically meaningful pathway activation differences; (2) is extensible to user-defined pathway coefficient tables; (3) distinguishes pathway-specific signals from random variation; (4) provides complementary information beyond expression-based scoring methods; and (5) is robust to common preprocessing choices and distance metric specifications, with normalized inputs recommended for routine use.

###

**Supplementary Figure 4. Sensitivity Test** (A) Density plot and (B) outcomes of PathwayEmbed using different input matrix type with or without scale. (C) Correlation between score and dataset technical quality using raw and normalized counts. (D) Correlation between scores generated using Manhanttan vs Eucildean distance metric.

**Validation of Wnt pathway using be-catenin KO system**

To validate PathwayEmbed against a known biological ground truth, we applied it to a public dataset (GEO accession: GSE233979) which represents a beta-catenin perturbed cell system (Hua et al. 2024). In this system, disruption of Wnt signaling via β-catenin (Ctnnb1) enhancer knockout is expected to reduce canonical WNT pathway activity, providing a well-defined biological ground truth for validation. We applied the same four condition-specific WNT pathway coefficient tables used in the synthetic dataset benchmarking above (WNT3A_12H, WNT3A_24H, WNT3A_48H, and WNT3A_SLOPE), all derived from the external GSE103175 perturbation dataset and therefore entirely orthogonal to the validation data. PathwayEmbed consistently detected differences in pathway activity between KO and WT cells across all pathway coefficient tables. The slope-based pathway coefficients yielded the most pronounced separation (Cohen’s d ≈ −0.301). Unlike the timepoint coefficient tables where coefficients are assigned based on the direction of differential expression at a single timepoint relative to the untreated baseline, the slope coefficients are assigned based on the sign of a gene-wise linear regression slope computed across all four timepoints (0, 12, 24, and 48 hours). Genes with a consistently increasing trajectory across the time course receive a coefficient of +1 and those with a consistently decreasing trajectory receive −1, capturing sustained monotonic trends rather than snapshot responses. Therefore, the prominent differences observed there suggest that the knockout system reflects a more stabilized or sustained transcriptional shift rather than an acute early response to pathway perturbation **(Supplementary Fig. 5A)**. This result validates the PathwayEmbed in known system and emphasizes the utility of condition-specific coefficient tables to capture context-dependent signaling behavior.

To assess whether pathway activity scores were influenced by technical factors, we examined correlations between PathwayEmbed scores and commonly used quality metrics, including total transcript counts (nCount), number of detected genes (nFeature), and mitochondrial gene percentage (percent.mt). PathwayEmbed scores showed minimal correlation with these metrics, indicating that inferred pathway activity is largely independent of sequencing depth and cell quality **(Supplementary Fig. 5B).**

We next benchmarked PathwayEmbed against alternative pathway and expression-based scoring approaches, including PROGENy, AddModuleScore, mean expression, and z-scored mean expression. Consistent with the known biological ground truth that β-catenin knockout reduces WNT signaling, PathwayEmbed detected decreased pathway activity in KO cells and demonstrated stronger effect sizes and classification performance compared to PROGENy, likely due to the different scoring strategies **(Supplementary Fig. 5C)**. Specifically, PROGENy scores are computed using a model trained on a large compendium of bulk perturbation experiments but lack inherent bounds, whereas PathwayEmbed generates scores that are normalized to ON and OFF references within the input dataset.

However, expression-based aggregation methods (mean and z-scored expression) showed higher correlation with pathway scores and, in some cases, stronger classification performance, indicating that global transcriptional differences contribute substantially to signal detection in this dataset. In contrast, PathwayEmbed exhibited weaker correlation with these expression-based metrics, suggesting that it captures pathway-specific structure that is partially independent of overall expression magnitude.

Together, these results demonstrate that PathwayEmbed can recover biologically expected signaling changes in a real perturbation system while maintaining robustness to technical confounding factors.

**Supplementary Figure 5. Application of PathwayEmbed in known b-catenin KO system** (A) Density plot and (B) outcomes of PathwayEmbed using different input matrix type with or without scale. (C) Correlation between score and dataset technical quality using raw and normalized counts. (D) Correlation between scores generated using Manhanttan vs Eucildean distance metric.

**Validation of Notch signaling pathway in Notch perturbed system and aging cells.**

Notch signaling is another important signaling pathway regulating cell fate and tissue functions. To select an appropriate Notch pathway coefficient table, we evaluated multiple perturbation-derived bulk-RNA seq datasets and analyzed both activation and inhibition paradigms across human and mouse systems. For inhibition-based datasets, γ-secretase inhibitors CB-103 and LY411575 were applied to human RPMI-8402 T-ALL cells (GSE221577) (Cao et al. 2023). For activation-based datasets, recombinant JAG1 protein was used to stimulate mouse embryonic endocardial cells (GSE223735)(Luna-Zurita et al. 2023) and human SVG-A astrocyte-derived cells across a 2-, 4-, and 24-hour time course (GSE235637)(Tveriakhina et al. 2024), yielding condition-specific pathway coefficient tables that capture early and sustained NOTCH transcriptional responses.

To validate the framework, we then applied PathwayEmbed to skeletal stem/progenitor cells (SSPCs) from Notch KO and WT marrow (GEO accession: GSE240292) (Remark et al. 2023) to evaluate their Notch transduction state. The initial data process followed the protocol in the original publication and SSPCs are identified using markers reported (Remark et al. 2023). Because this dataset derives from a mouse genetic knockout model, we first prioritized the mouse-based NOTCH pathway coefficient table generated from recombinant JAG1 stimulation of endocardial cells (GSE223734). We additionally evaluated the human 24-hour JAG1 activation database (GSE235637) as a cross-species comparison. Interestingly, despite the species mismatch, the 24-hour activation pathway coefficient table produced stronger separation between KO and WT SSPCs **(Supplementary Fig. 6A)**, likely attributable to its greater gene coverage and inclusion of additional pathway-informative genes that enhance signal detection. Importantly, shared genes between the two pathway coefficient tables exhibited consistent coefficient directions across species, supporting the biological robustness of the inferred pathway activity. Consistent with improved gene coverage, PathwayEmbed scores derived from the 24-hour pathway coefficient table also showed stronger correlations with alternative scoring methods including AddModuleScore, mean expression, and z-scored expression **(Supplementary Fig. 6B)**. Across both pathway coefficient table choices, PathwayEmbed outperformed AddModuleScore and z-score-based approaches in both effect size (Cohen's d) and classification performance (AUROC), demonstrating that incorporating gene-specific directionality and empirically derived coefficients provides a meaningful advantage over unweighted gene set scoring methods for pathway activity inference **(Supplementary Fig. 6D)**.

**Supplementary Figure 6. Validation of Notch Pathway Analysis** (A) Density plot of Notch transduction states using mouse and human datasets. (B) Correlation analysis between different metrics (C) Effect size and AUROC of KO vs WT separation using different measurements.

Given the robust performance of the Notch 24hr pathway dataset, we proceeded analyze young versus middle-aged SSPCs from Remark 2023(Remark et al. 2023). Our analysis revealed a significant increase in Notch pathway activity in middle-aged SSPCs compared to young cells (**Supplementary Fig 3F**), consistent with published findings (Remark et al. 2023) and providing independent validation of the PathwayEmbed approach.

To more broadly characterize the signaling landscape of skeletal aging, we extended the analysis to four additional pathways, Hippo/YAP, HIF-1α, TGF-β, and WNT. For the Hippo/YAP pathway, the pathway coefficient table was derived from heat stress–induced YAP activation in mouse melanoma cells (GSE133251)(Luo et al. 2020). Because heat stress activates YAP while suppressing the upstream Hippo kinase cascade, coefficient polarity was inverted so that +1 consistently denotes expected expression during canonical Hippo pathway activation. The HIF-1α pathway coefficient table was constructed from hypoxia-induced activation (1% O₂) of primary human bronchial epithelial cells (GSE227502)(Mikami et al. 2023) across three timepoints (6 hours, 24 hours, and 5 days), capturing the physiological transcriptional response to oxygen deprivation without pharmacological or genetic perturbation. For TGF-β, the pathway coefficient table derived from TGF-β1 stimulation of mouse T cells (GSE246932)(Taber et al. 2023) was selected over the one based on human fibroblast datasets (Walker et al. 2019) to maximize species consistency with the mouse SSPC data. For WNT, the time-slope pathway coefficient table which was previously validated in the Wnt knockout benchmarking experiment was utilized in this case again.

Except for the Hippo/YAP pathway, which showed reduced transduction activity in middle-aged SSPCs, all other pathways including NOTCH, HIF-1α, TGF-β, and WNT exhibited increased activation in the middle-aged group relative to young cells **(Supplementary Fig. 7A)**. Quantitative comparison using Cohen's d **(Supplementary Fig. 7B)** and AUROC (shown in vignette) confirmed that NOTCH produced the strongest effect, further underscoring the established role of Notch signaling in aging-related transcriptional reprogramming of skeletal progenitors.

To assess whether these pathway activity scores reflect genuine biological signaling rather than technical or cellular confounders, we examined correlations between pathway scores and a panel of quality and cell-state variables, including sequencing depth, gene complexity, mitochondrial content, cell cycle phase, and stress-related gene expression. Pathway scores showed minimal correlation with all tested variables, indicating that the inferred activity levels are largely independent of cell quality metrics and general cellular state **(Supplementary Fig. 7C)**. Finally, cross-pathway correlation analysis revealed limited interdependency among the five pathway scores **(Supplementary Fig. 7D)**, suggesting again that each captures a largely distinct transcriptional program rather than a shared global axis of transcriptional change. Collectively, these results support that the multi-pathway activity landscape revealed by PathwayEmbed reflects genuine, pathway-specific signaling alterations accompanying skeletal aging.

**Supplementary Figure 7. Application of PathwayEmbed to middle-age vs young cells.** (A) Analysis of SSPCs from young versus middle-aged mice using pathwaybed, showing increased Notch pathway activation in middle-aged cells. (B) Effect size (Cohen’s d) across pathways. Spearman correlation (C) between each pathway and technical cofounders and (D) among each pathway.

**Application of PathwayEmbed in mouse developing embryo spatial data**

To further illustrate its application, we applied PathwayEmbed to a mouse orogenesis embryo spatial data collected from E9.5 to E12.5 (Chen et al. 2022). This spatial dataset captures organogenesis at high resolution over time. We were interested in estimating spatial distributions of signal transduction through the Wnt, Notch, Tgfb, Hippo, and HIF-1a pathways during tissue development. Each pathway coefficient table is constructed using the method described before.

Score calculation and normalization were computed globally to facilitate direct comparisons between development timepoints (**Supplementary Fig. 8A**). Temporal comparison of the proportion of pathway-active spots revealed divergent trajectories across the five pathways. For WNT, NOTCH, TGF-β, and HIF-1α, the proportion of pathway-active spots generally declined from E9.5 to E12.5. In the contrast, Hippo/YAP pathway showed an increase in active spots across development. These temporal trends were statistically significant across timepoints for all five pathways (Kruskal-Wallis test, all p < 0.05; **Supplementary Fig. 9A**).

Furthermore, all five inferred pathway activities exhibited significant positive spatial autocorrelation (Moran's I range from 0.086 to 0.255, all p ≈ 0) (**Supplementary Fig. 8B)**, indicating that pathway transduction states are spatially structured rather than randomly distributed across the embryo. HIF-1α and NOTCH showed the strongest spatial autocorrelation (I = 0.255 and 0.211, respectively), followed by TGF-β (I = 0.126), Hippo (I = 0.093), and WNT (I = 0.086). Local Moran's I analysis of the two highest-autocorrelation pathways, NOTCH and HIF-1α, revealed spatially coherent high–high and low–low clusters (**Supplementary Fig. 8B)**, mimicking anatomically defined regions and highlighting the importance of the signaling axis in embryo development.

**Supplementary Figure 8. Application of PathwayEmbed to mouse embryo spatial data** (A) Spatial plots of different embryo compartments and Wnt, Tgfb, Notch, Hippo, and HIF-1a signaling transduction score. (B) Moran’s I of all pathways and (D) plot of local Moran’s I clusters for HIF-1a and Notch.

Cross-pathway comparison revealed largely independent signaling programs: pairwise Spearman correlations between all five pathway scores were modest (|ρ| ≤ 0.118), with only weak positive associations observed between HIf1α–NOTCH (ρ = 0.118) and TGF-β–NOTCH (ρ = 0.099), and a weak negative association between Hippo and TGF-β (ρ = −0.093) (**Supplementary Fig. 9B**). This mutual independence indicates that each pathway captures a largely distinct spatial transcriptional program rather than a shared global axis of gene expression.

Comparison with PROGENy scores for the three overlapping pathways revealed a gradient of agreement (**Supplementary Fig. 9D)**. HIF-1α showed the strongest positive correlation with PROGENy Hypoxia (Spearman ρ = 0.480), followed by TGF-β vs. PROGENy TGFb (ρ = 0.294), and WNT vs. PROGENy WNT (ρ = 0.051). The moderate-to-strong correlation for HIF-1α and TGF-β suggests meaningful overlap in the gene programs captured by both tools, while the markedly lower agreement for WNT likely reflects the fundamentally different pathway coefficient table construction and scoring strategies discussed before.

Analysis of correlations between pathway scores and potential technical and biological confounders revealed an informative pattern (**Supplementary Fig. 9C)**. All pathway scores showed statistically significant but generally weak correlations with sequencing depth (nCount) and gene complexity (nFeature). However, the magnitude of these correlations mirrored the Moran's I ranking: HIF-1α and NOTCH, which had the highest spatial autocorrelation, also showed the strongest associations with nCount (ρ = 0.421 and 0.308, respectively), cell cycle S-phase score (ρ = 0.380 and 0.275), and stress score (ρ = 0.247 and 0.169). WNT, by contrast, showed minimal correlation with all confounders (|ρ| ≤ 0.087). Importantly, this parallel ranking suggests that HIF-1α and NOTCH scores partially reflect a gradient of cellular metabolic activity and proliferative state across the embryo rather than purely technical noise. Consistent with this interpretation, all pathways showed negligible correlation with the cell cycle combined score (CC_score: |ρ| ≤ 0.026, non-significant for Hippo and TGF-β), and pathway scores were mutually independent, arguing against a single global confounding axis.

Together, these findings support that the spatial signaling landscape inferred by PathwayEmbed reflects genuine pathway-specific biology distributed across the developing embryo, with local coherence of signal transduction density becomes clearer at later time points.

**Supplementary Figure 9. Application of PathwayEmbed to mouse embryo spatial data.** (A) Density plots of above pathway transduction states across different time points. (B) Spearman correlation between individual pathways

## **Discussion**

The PathwayEmbed package introduces a novel framework for quantifying and visualizing pathway activation states at single-cell resolution by integrating curated pathway molecule coefficients with gene expression data. Unlike many existing pathway analysis tools that primarily focus on gene set enrichment or pathway-level differential expression, PathwayEmbed offers a continuous, cell-by-cell transduction score that reflects both the expression magnitude and the directional influence (activating or inhibitory) of individual pathway components. The core innovation lies in positioning each cell geometrically between a dataset derived ON state, representing maximal expected pathway activity given the molecules and their directionalities, and a corresponding OFF state. This nuanced approach allows for a more mechanistic interpretation of pathway activity dynamics, moving beyond binary or categorical classifications.

Traditional pathway analysis tools such as GSEA and AddModuleScore treat pathway gene sets as unweighted collections and summarize activity through aggregate expression. This makes them sensitive to global transcriptional shifts but blind to the directionality with which individual genes contribute to signal transduction. PROGENy partially addresses this limitation by incorporating perturbation-derived gene weights from bulk RNA-seq footprinting, providing more mechanistically informed gene signatures. However, PROGENy’s weights and scoring system are fixed across contexts. In contrast, PathwayEmbed takes a fundamentally different approach: rather than computing an absolute summary score, it generates scores based on ON and OFF anchor states derived directly from the dataset under analysis. Specifically, PathwayEmbed first identifies the expression extremes of each pathway gene within the input data weighted by their assigned directional coefficients. Each cell is then scored by its distance to these two data-derived poles, normalized within the global observed range. This design ensures that the resulting score reflects a cell's position within the signaling space as it exists in the dataset being analyzed, rather than relative to a population that may have been profiled under entirely different conditions. In this sense, PathwayEmbed is most precisely understood as a within-dataset relative positioning tool rather than an absolute pathway activity meter.

This design choice, however, has important implications for cross-dataset and cross-pathway comparisons. Because ON and OFF anchors are recomputed independently for each dataset, scores are not directly comparable across experiments. A score of 0.7 in one dataset does not necessarily correspond to the same level of pathway activity in another. Similarly, comparing scores across pathways within a single dataset (e.g., concluding that a cell exhibits “more WNT activity than NOTCH activity”) is not straightforward, as pathways differ in gene set composition, size, and expression scale, and their anchors are calibrated independently. This distinguishes PathwayEmbed from methods such as PROGENy, which rely on a fixed reference and therefore produce scores that are more directly comparable across datasets and pathways. Accordingly, PathwayEmbed scores should be interpreted primarily within a single dataset, where they are well suited for comparing cell populations, time points, or spatial regions. Developing principled approaches for cross-dataset normalization represents an important direction for future work.

Another area for future improvement is the construction of pathway coefficient tables. In the current implementation, coefficients encode directionality only (+1 or −1) and not differential weighting between genes. This is a deliberate simplification reflecting the present state of mechanistic knowledge: for most pathway components, well-validated, context-independent estimates of the quantitative magnitude of transcriptional response to activation do not yet exist. Equal weighting therefore avoids introducing unsupported assumptions that could bias results. Nonetheless, incorporating gene-specific weights represents a key opportunity for improving biological fidelity in future versions.

Beyond cross-dataset comparability and coefficient weighting, several additional limitations should be noted. Pathway activity scores reflect relative activity within the analyzed dataset rather than absolute activation levels. The current coefficient table covers only five pathways, requiring users working beyond this set to construct their own gene–coefficient mappings, as described in the accompanying vignettes. Additionally, pathway crosstalk, cell-type-specific regulatory rewiring, and post-transcriptional regulation are not fully captured by the current model.

In summary, PathwayEmbed provides a flexible framework for signaling transduction state estimation. Further efforts in signaling pathway dataset development will allow a more accurate translation from high-through multi-omics to realistic biological insights.

**References**

Cao L, Ruiz Buendia GA, Fournier N, Liu Y, Armand F, Hamelin R, Pavlou M, Radtke F (2023) Resistance mechanism to Notch inhibition and combination therapy in human T-cell acute lymphoblastic leukemia. Blood Adv 7 (20):6240-6252. doi:10.1182/bloodadvances.2023010380

Chen A, Liao S, Cheng M, Ma K, Wu L, Lai Y, Qiu X, Yang J, Xu J, Hao S, Wang X, Lu H, Chen X, Liu X, Huang X, Li Z, Hong Y, Jiang Y, Peng J, Liu S, Shen M, Liu C, Li Q, Yuan Y, Wei X, Zheng H, Feng W, Wang Z, Liu Y, Wang Z, Yang Y, Xiang H, Han L, Qin B, Guo P, Lai G, Munoz-Canoves P, Maxwell PH, Thiery JP, Wu QF, Zhao F, Chen B, Li M, Dai X, Wang S, Kuang H, Hui J, Wang L, Fei JF, Wang O, Wei X, Lu H, Wang B, Liu S, Gu Y, Ni M, Zhang W, Mu F, Yin Y, Yang H, Lisby M, Cornall RJ, Mulder J, Uhlen M, Esteban MA, Li Y, Liu L, Xu X, Wang J (2022) Spatiotemporal transcriptomic atlas of mouse organogenesis using DNA nanoball-patterned arrays. Cell 185 (10):1777-1792 e1721. doi:10.1016/j.cell.2022.04.003

Hua X, Zhao C, Tian J, Wang J, Miao X, Zheng G, Wu M, Ye M, Liu Y, Zhou Y (2024) A Ctnnb1 enhancer transcriptionally regulates Wnt signaling dosage to balance homeostasis and tumorigenesis of intestinal epithelia. Elife 13. doi:10.7554/eLife.98238

Huggins IJ, Bos T, Gaylord O, Jessen C, Lonquich B, Puranen A, Richter J, Rossdam C, Brafman D, Gaasterland T, Willert K (2017) The WNT target SP5 negatively regulates WNT transcriptional programs in human pluripotent stem cells. Nat Commun 8 (1):1034. doi:10.1038/s41467-017-01203-1

Luna-Zurita L, Flores-Garza BG, Grivas D, Siguero-Alvarez M, de la Pompa JL (2023) Cooperative Response to Endocardial Notch Reveals Interaction With Hippo Pathway. Circ Res 133 (12):1022-1039. doi:10.1161/CIRCRESAHA.123.323474

Luo M, Meng Z, Moroishi T, Lin KC, Shen G, Mo F, Shao B, Wei X, Zhang P, Wei Y, Guan KL (2020) Heat stress activates YAP/TAZ to induce the heat shock transcriptome. Nat Cell Biol 22 (12):1447-1459. doi:10.1038/s41556-020-00602-9

Mikami Y, Grubb BR, Rogers TD, Dang H, Asakura T, Kota P, Gilmore RC, Okuda K, Morton LC, Sun L, Chen G, Wykoff JA, Ehre C, Vilar J, van Heusden C, Livraghi-Butrico A, Gentzsch M, Button B, Stutts MJ, Randell SH, O'Neal WK, Boucher RC (2023) Chronic airway epithelial hypoxia exacerbates injury in muco-obstructive lung disease through mucus hyperconcentration. Sci Transl Med 15 (699):eabo7728. doi:10.1126/scitranslmed.abo7728

Remark LH, Leclerc K, Ramsukh M, Lin Z, Lee S, Dharmalingam B, Gillinov L, Nayak VV, El Parente P, Sambon M, Atria PJ, Ali MAE, Witek L, Castillo AB, Park CY, Adams RH, Tsirigos A, Morgani SM, Leucht P (2023) Loss of Notch signaling in skeletal stem cells enhances bone formation with aging. Bone Res 11 (1):50. doi:10.1038/s41413-023-00283-8

Taber A, Konecny A, Oda SK, Scott-Browne J, Prlic M (2023) TGF-beta broadly modifies rather than specifically suppresses reactivated memory CD8 T cells in a dose-dependent manner. Proc Natl Acad Sci U S A 120 (48):e2313228120. doi:10.1073/pnas.2313228120

Tveriakhina L, Scanavachi G, Egan ED, Da Cunha Correia RB, Martin AP, Rogers JM, Yodh JS, Aster JC, Kirchhausen T, Blacklow SC (2024) Temporal dynamics and stoichiometry in human Notch signaling from Notch synaptic complex formation to nuclear entry of the Notch intracellular domain. Dev Cell 59 (11):1425-1438 e1428. doi:10.1016/j.devcel.2024.03.021

Walker EJ, Heydet D, Veldre T, Ghildyal R (2019) Transcriptomic changes during TGF-beta-mediated differentiation of airway fibroblasts to myofibroblasts. Sci Rep 9 (1):20377. doi:10.1038/s41598-019-56955-1
